# Utility of Transient Elastography for the Screening of Liver Disease in Patients with Alpha1-Antitrypsin Deficiency

**DOI:** 10.3390/jcm10081724

**Published:** 2021-04-16

**Authors:** Mònica Pons, Alexa Núñez, Cristina Esquinas, María Torres-Durán, Juan Luis Rodríguez-Hermosa, Myriam Calle, Ramón Tubio-Pérez, Irene Belmonte, Francisco Rodríguez-Frías, Esther Rodríguez, Joan Genescà, Marc Miravitlles, Miriam Barrecheguren

**Affiliations:** 1 Liver Unit, Department of Internal Medicine, Hospital Universitari Vall d’Hebron, Vall d’Hebron Institut de Recerca (VHIR), Vall d’Hebron Barcelona Hospital Campus, 08035 Barcelona, Spain; monica_xina@hotmail.com (M.P.); jgenesca@vhebron.net (J.G.); 2 Pneumology Department, Hospital Universitari Vall d’Hebron/Vall d’Hebron Institut de Recerca (VHIR), Vall d’Hebron Barcelona Hospital Campus, 08035 Barcelona, Spain; alexagnd01@hotmail.com (A.N.); crise4@hotmail.com (C.E.); irbelmu@gmail.com (I.B.); estherod@vhebron.net (E.R.); mbarrech@vhebron.net (M.B.); 3 Department of Medicine, Universitat Autònoma de Barcelona, 08193 Barcelona, Spain; 4 Pneumology Department, University Hospital Complex of Vigo, Instituto de Investigación Biomédica Galicia Sur, 36213 Vigo, Spain; mtordur@yahoo.es (M.T.-D.); ramon.antonio.tubio.perez@sergas.es (R.T.-P.); 5 Pneumology Department, Instituto de Investigación Sanitaria del Hospital Clínico San Carlos (IdISSC), Hospital Clínico de San Carlos, Departamento de Medicina, Facultad de Medicina, Universidad Complutense de Madrid, 28040 Madrid, Spain; jlrhermosa@yahoo.es (J.L.R.-H.); mcallerubio@gmail.com (M.C.); 6 Department of Clinical Biochemistry, Hospital Universitari Vall d’Hebron, Vall d’Hebron Barcelona Hospital Campus, 08035 Barcelona, Spain; frarodri@gmail.com; 7 Centro de Investigación Biomédica en Red de Enfermedades Hepáticas y Digestivas (CIBEREHD), 28029 Madrid, Spain; 8 Centro de Investigación Biomédica en Red de Enfermedades Respiratorias (CIBERES), 28029 Madrid, Spain

**Keywords:** alpha1-antitrypsin deficiency, liver disease, transient elastography

## Abstract

Screening of liver disease in alpha-1 antitrypsin deficiency (AATD) is usually carried out with liver enzymes, with low sensitivity. We conducted a multicenter cross-sectional study aiming to describe the utility of transient elastography for the identification of liver disease in patients with AATD. A total of 148 AATD patients were included. Among these, 54.7% were Pi*ZZ and 45.3% were heterozygous for the Z allele. Between 4.9% and 16.5% of patients had abnormal liver enzymes, without differences among genotypes. Liver stiffness measurement (LSM) was significantly higher in Pi*ZZ individuals than in heterozygous Z (5.6 vs. 4.6 kPa; *p* = 0.001). In total, in 8 (5%) individuals LSM was >7.5 kPa, considered significant liver fibrosis, and ≥10 kPa in 3 (1.9%) all being Pi*ZZ. Elevated liver enzymes were more frequently observed in patients with LSM > 7.5 kPa, but in 5 out of 8 of these patients all liver enzymes were within normal range. In patients with AATD, the presence of abnormal liver enzymes is frequent; however, most of these patients do not present significant liver fibrosis. Transient elastography can help to identify patients with liver fibrosis even with normal liver enzymes and should be performed in all Z-allele carriers to screen for liver disease.

## 1. Introduction

Alpha1-antitrypsin deficiency (AATD) is caused by a specific mutation of the SERPINA 1 gene which results in abnormal production and low circulating levels of alpha1-antitrypsin (AAT). It is one of the most common genetic diseases in adulthood and is associated with an increased risk of developing pulmonary emphysema and liver disease [1,2].

AAT is a protein synthesized and secreted mainly by hepatocytes, the main function of which is to protect lung tissue from damage caused by proteolytic enzymes such as neutrophil elastase [2]. AAT is a highly polymorphic protein with more than 120 variants, including about 60 deficient alleles. The normal allele, present in more than 95% of normal subjects, is called M [1,2]. The most frequent deficient alleles are S and Z, and they are found in 10% and 2% of the Spanish population, respectively [3,4,5,6].

The Z variant presents an alteration in its tertiary structure that facilitates misfolding of the protein and gives rise to the spontaneous formation of polymers, leading to the accumulation of the protein in the endoplasmic reticulum of the hepatocytes [7,8]. Liver damage is then caused by this protein accumulation, inducing apoptosis of the hepatocytes and a compensatory hepatocyte proliferation that eventually produces liver fibrosis that can evolve to cirrhosis or hepatocellular carcinoma [8,9]. Most patients with liver disease are homozygous for the deficient Z allele (Pi*ZZ), although different degrees of liver involvement have been described in heterozygotes (Pi*SZ and Pi*MZ), especially if associated with other co-factors such as alcohol consumption or metabolic syndrome [10,11].

Currently there is no non-invasive gold standard technique for the screening and early diagnosis of liver disease in patients with AATD [12]. In clinical practice, liver enzymes are routinely checked, while liver ultrasound is performed if necessary. However, it has been observed that transaminase levels have a low sensitivity to identify liver disease, and they correlate little with the degree of liver disease, especially in adulthood [13]. Serum biomarkers and image devices based on elastography technique have been developed to overcome this problem and to assess the presence of fibrosis in liver diseases of different etiologies [14,15].

Recently, there has been increasing interest in the use of elastographic methods, such as transient elastography, for screening liver disease in AATD patients [16,17,18]. However, the screening and management of asymptomatic liver disease in AATD can differ among centers due to a lack of consensus or guidelines. Therefore, the aim of our study was to describe the utility of transient elastography for the identification of liver disease in patients with AATD.

## 2. Materials and Methods

This was a multicenter cross-sectional study including patients older than 18 years with mild, moderate, and severe AATD (Pi*MS, SS, MZ, SZ, ZZ, and rare variants) consecutively recruited from the outpatient Pneumology Clinics of three AATD reference centers in Spain (Vall d’Hebron University Hospital, Barcelona, University Hospital Complex of Vigo, and Hospital Clínico San Carlos, Madrid) from 1 April 2017 to 1 January 2020. As part of the assessment of patients with AATD, all of them were offered blood analysis, full lung function tests, and transient elastography, and the only exclusion criterion was to refuse to sign informed consent. The study was approved by the Vall d’He- bron Hospital Ethics Committee (Barcelona, Spain), number PR(AG)335/2016, and all patients provided written informed consent.

### 2.1. Variables

During the first visit, a complete physical examination was performed in all patients with special interest in signs of chronic liver disease such as splenomegaly, jaundice, or palmar erythema. Sociodemographic and clinical characteristics were collected and other parameters such as body mass index (BMI), lung function tests (forced expiratory volume in the first second (FEV1), FEV1/forced ventilatory capacity (FVC), and carbon monoxide transfer coefficient (KCO)), comorbidities, treatments, and AAT augmentation therapy were reported. Diagnosis of chronic obstructive pulmonary disease (COPD) was established when the post-bronchodilator FEV1/FVC ratio was below 0.7.

Blood samples were obtained for determination of liver function tests: Aspartate aminotransferase (AST), alanine aminotransferase (ALT), gamma-glutamyl transferase (GGT), alkaline phosphatase (ALP), international normalized ratio (INR), platelet count, and albumin. In addition, the Fibrosis-4 (FIB-4) score was calculated as age (years) × AST [IU/L]/(platelet count [109/L] × √ALT [IU/L]) and AST-to-platelet ratio index (APRI) as (AST [IU/L]/40 IU/L)/platelet count [109/L] × 100. Patients were classified according to the previously established FIB-4 cut-offs of <1.45 with a high negative predictive value for ruling out advanced fibrosis and >3.25 with a high specificity and a 65% positive predictive value for ruling in advanced fibrosis [19]. For APRI, we used the cut-off <0.5 for excluding cirrhosis (high negative predictive value) and >1.0 as a high specific cut-off for predicting cirrhosis [20].

The Enhanced Liver Fibrosis (ELF) test (Siemens Healthcare Diagnostics, Vienna, Austria) was available as a biomarker of liver fibrosis in one of the centers. The ELF test is a panel of markers that consists of 3 components: Type III procollagen peptide, hyaluronic acid, and tissue inhibitor of metalloproteinase-1. We explored the manufacturer- recommended 9.8 cut-off to rule in advanced fibrosis [21].

### 2.2. Liver Stiffness Measurement by Transient Elastography

Liver stiffness measurements (LSM) were performed in a fasting state using a Fibroscan 502 Touch (Echosens, Paris, France) using the M or XL probe as per device indication. Quality criteria used in all centers were at least 10 valid measurements and an interquartile-to-median ratio ≤ 30%. The LSM technique was carried out in accordance with the European Association for Study of the Liver (EASL) clinical guidelines [22].

Results were expressed in kilopascals (kPa). Normal liver stiffness values are around 5 kPa. Transient elastography has good re-producibility and has good diagnostic performance for estimating liver fibrosis. However, the accuracy is not as good for detecting significant fibrosis compared to advanced fibrosis or cirrhosis [22,23]. Since there are no specific LSM cut-offs for AATD liver disease, a LSM > 7.5 kPa was used as suggestive of significant fibrosis and ≥10 kPa was suggestive of advanced fibrosis according to previously established cut-offs in other liver diseases (mainly viral etiologies and alcoholic liver disease) [22,24].

The presence of steatosis was assessed by the controlled attenuation parameter (CAP) and results were expressed in decibel per meter (dB/m). The cut-off >268 dB/m was used as an indicator of moderate steatosis, and for severe steatosis the cut-off was >280 dB/m [25].

### 2.3. Statistical Analysis

Qualitative variables were described with absolute frequencies and percentages. The description of quantitative variables was performed using the mean, standard deviation (SD) or median, and interquartile range (IQR). The Kolmogorov–Smirnov test was used to assess the normality of distributions.

Patient characteristics were compared according to genotypes and other clinical conditions. In the case of quantitative variables, the Student’s *t*-test for normally distributed variables or the Mann–Whitney U-test if normality was not assumed was used, while ANOVA tests were performed in the case of variables with more than 2 categories. The Chi-squared test (Fisher test for frequencies < 5) was used for the comparison of categorical variables. A linear relationship between quantitative variables, in particular between surrogates of liver disease (LSM, CAP and FIB-4) and spirometric markers of airflow obstruction (FEV1(%) and FEV1/FVC), were analyzed using Spearman tests. For all the tests, *p*-values < 0.05 were considered statistically significant. The statistical package R Studio (V2.5.1) was used for the analyses.

## 3. Results

### 3.1. Demographic and Clinical Findings

A total of 148 AATD patients were included from January 2017 to December 2019. Among these, 81 (54.7%) were homozygous Pi*ZZ and 67 (45.3%) were heterozygous for the Z allele (29 Pi*SZ, 35 Pi*MZ, 1 Pi*FZ, 1 Pi*PlowellZ, 1 Pi*MmaltonZ).

The mean age was 52.5 and 57 years for heterozygous and Pi*ZZ, respectively, and 50% of the patients were male. Liver disease in infancy was reported as the cause of the diagnosis of AATD in 19.4% and 11.1% of heterozygous and homozygous patients, although there were no patients with an active diagnosis of liver disease at the time of the study. COPD was diagnosed in 22.7% of heterozygous subjects and up to 70% for Pi*ZZ patients. Consequently, the mean FEV1 (%) was significantly lower in Pi*ZZ compared with heterozygous (69% (SD: 30.5%) versus 92.9% (SD: 27.6%); *p* < 0.001). The baseline characteristics of the global population and the two genotype groups are shown in Table 1.

### 3.2. Clinical and Laboratory Signs of Liver Disease

Thirty-two patients (21.6%) had abnormal liver enzymes. The distribution of values showed significant differences only in AST values, which were significantly higher in Pi*ZZ patients (29.2 UI/L (SD: 15.4) vs. 25.0 UI/L (SD: 8.0; *p* = 0.029). The most frequent pattern was an elevation in GGT (14.9% of patients). Pi*ZZ patients had a higher FIB-4 score compared to heterozygous Z (1.6 (SD: 0.8) vs. 1.2 (SD:0.5); *p* < 0.001). Only 5 patients had FIB-4 > 3.25 and all were Pi*ZZ. The APRI score was higher in Pi*ZZ patients than in heterozygous Z (0.35 (SD: 0.18) vs. 0.27 (SD: 0.09); *p* = 0.007), but most of the patients had APRI values < 0.5, excluding advanced fibrosis or cirrhosis, and only one Pi*ZZ patient had an APRI score > 1.0. The ELF score was obtained in 52 patients (27 Pi*ZZ and 25 Pi*Z patients). Pi*ZZ had significantly higher values compared to Pi*Z phenotypes (8.6 (SD: 0.8) vs. 8 (SD: 0.6); *p* = 0.007). Only 1 Pi*ZZ patient showed values above the cut-off of 9.8 (Table 2).

### 3.3. Transient Elastography

**Figure 1:**
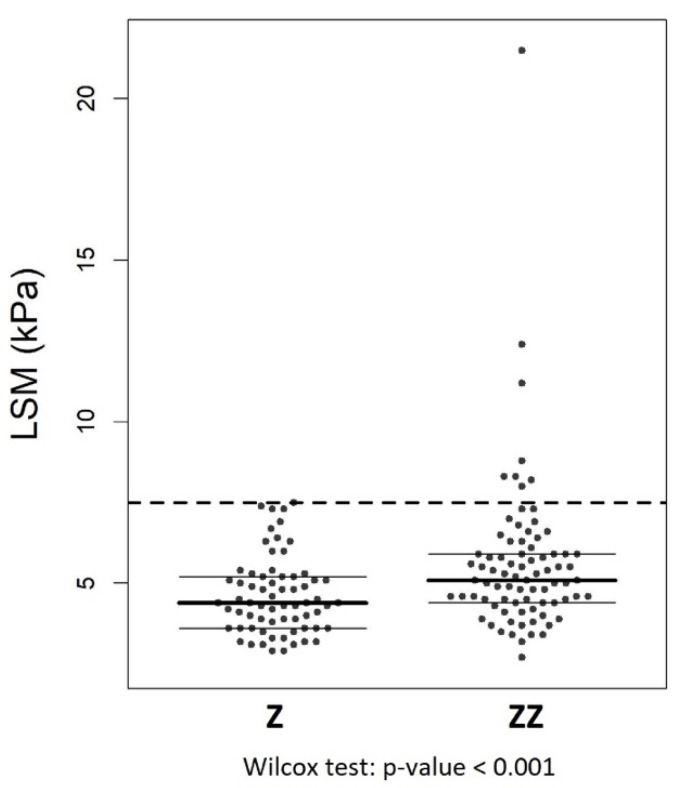
Comparison of mean LSM values by phenotype.

The mean LSM was significantly higher in Pi*ZZ individuals than in heterozygous Z (5.6 (SD: 2.5) kPa vs. 4.6 (SD:1.2) kPa, respectively; *p* = 0.007). In total, LSM was >7.5 kPa in 8 (5%) individuals and ≥10 kPa in 3 (1.9%), all being Pi*ZZ (Figure 1). By lowering the cut-off of LSM to >7.1 kPa as suggested in other studies [11], we found 10 Pi*ZZ patients (12.3%) and 3 heterozygous patients (4.5%), two of whom were Pi*SZ patients with LSM 7.3 kPa, and one was a Pi*MZ patient with LSM 7.5 kPa.

Using the LSM > 8.45 kPa cut-off of the study by Clark et al. [13], we would have identified 4 Pi*ZZ patients (4.9%) suggestive of having F ≥ 3.

Almost one-third of the patients had severe steatosis according to CAP values > 280 dB/m, with no significant differences between homozygous and heterozygous patients. (Table 2).

### 3.4. Characteristics of Pi*ZZ Patients According to LSM Values

Pi*ZZ individuals with LSM > 7.5 kPa were older and had a higher BMI. Two-thirds consumed alcohol, and all had COPD (versus 67% in patients with LSM ≤ 7.5 kPa; *p* = 0.097).

**Figure 2:**
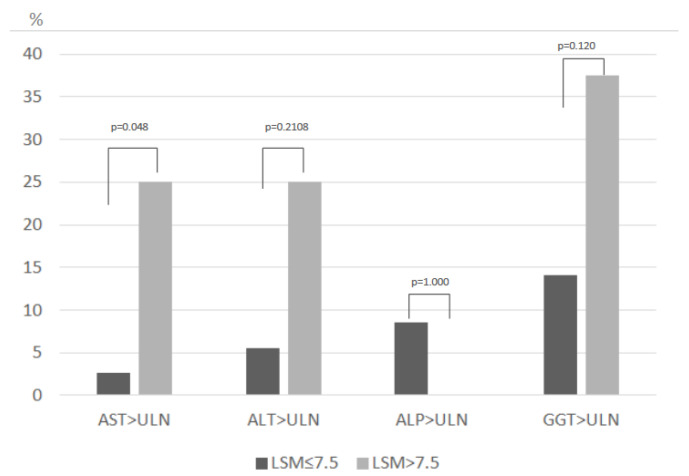
All individuals from the cohort with liver enzymes above the highest level of normal based on LSM values. UPN: Upper limit of normal for GGT: >38 IU/L in females and >55 IU/L in males.

Elevated liver enzymes were more frequently observed in patients with LSM > 7.5 kPa. Twenty-five percent of patients with LSM > 7.5 kPa had elevated AST values compared to 2.7% in patients with LSM ≤ 7.5 kPa (*p* = 0.048), and 37.5% of patients with LSM > 7.5 kPa had elevated GGT compared to 14.1% of patients with LSM ≤ 7.5 kPa (*p* = 0.120) (Table 3, Figure 2). Conversely, 11/61 patients (18%) had at least one elevated liver enzyme but with normal LSM values (LSM < 6 kPa). Correlations between LSM and liver enzymes were only significant, albeit weakly, between LSM and AST (0.311 (*p* < 0.001)), and LSM and GGT (0.389 (*p* < 0.001)).

**Figure 3:**
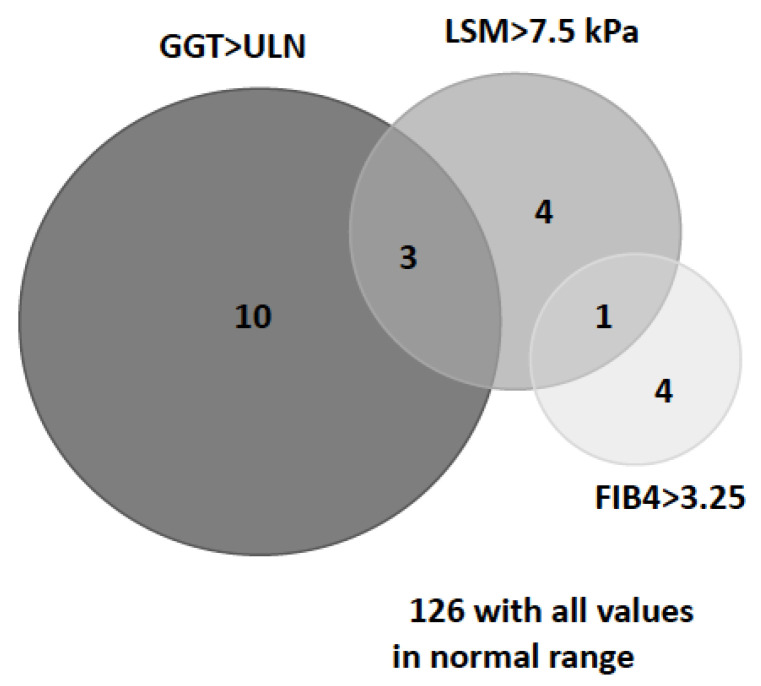
Relation between elevated GGT, FIB4, and LSM in Pi*ZZ patients. GGT: Gamma-glutamyl transferase; FIB-4: Fibrosis 4; LSM: Liver stiffness measurement; UPL: Upper limit of normal (according to sex-specific cut-offs: for GGT: >38 IU/L in females and >55 IU/L in males).

Among the 8 patients with LSM > 7.5, 3 had GGT above the normal limit and 1 also had a FIB-4 score > 3.25 (Figure 3). The FIB-4 score (2.2 (SD: 0.7) versus 1.5 (SD: 0.8); *p* = 0.032), as well as CAP measurement (317.9 (SD: 48) dB/m vs. 249.6 (SD: 56.5) dB/m; *p* = 0.004), were also higher in Pi*ZZ patients with LSM > 7.5 kPa (Table 3). Severe steatosis, with CAP > 280 dB/m, was present in 6 patients (75%) with LSM > 7.5 kPa compared to 20 patients (27.4%) with LSM < 7.5 kPa (*p* = 0.041).

The APRI was higher in Pi*ZZ patients with LSM > 7.5 kPa than in those with LSM ≤ 7.5 kPa (0.56 vs. 0.33, *p* < 0.001). The APRI had a significant correlation with LSM (r = 0.353, *p* = 0.030).

### 3.5. Comparison between Pi*ZZ Patients with or without COPD

Fifty-seven Pi*ZZ patients (70.4%) had COPD. Pi*ZZ patients with COPD were older and more frequently had a history of smoking compared with non-COPD individuals. As expected, they had worse lung function with a lower FEV1 (1.6 (SD: 0.8) L vs. 3.5 (SD: 1) L; *p* < 0.001) and KCO (%) (43.7% (SD: 30.8%) vs. 68% (SD: 30.4%); *p* = 0.003).

Regarding the liver study, no differences were observed in transaminase levels, but the FIB-4 score was higher in COPD patients (1.7 (SD: 0.8) vs. 1.2 (SD: 0.8); *p* = 0.046). More individuals in the COPD group had a LSM > 7.5 kPa (14% vs. 0%; *p* = 0.097) and they also had higher CAP values (265.9 (SD: 58.3) dB/m vs. 233.5 (SD: 55.8) dB/m; *p* = 0.023) (Table 3). Significant, albeit weak, correlations were found between FIB-4 and FEV1 (mL) (r = −0.350, *p* = 0.002), and CAP and FEV1 (mL) and FEV1(%) (r = −0.391, *p* < 0.001 and r = −0.306, *p* = 0.006, respectively). No significant correlations were found between LSM or ELF and measures of airflow obstruction.

## 4. Discussion

In our study population, we found that 10% of Pi*ZZ individuals had transient elastography results suggestive of liver fibrosis, but none of the heterozygous individuals reached the suggested threshold. Although individuals with higher LSM had higher transaminase levels and FIB-4 scores, normal levels of these biomarkers did not reliably rule out liver disease, since some of the patients with normal values had high LSM values. All patients with high LSM also had COPD.

Transient elastography is a non-invasive tool that has proven to be useful in the diagnosis of liver fibrosis of different etiologies. More recently, its utility has also been explored in AATD-related liver disease with promising results [16,17,18,26]. Although different cut-offs have been proposed, there is no validated cut-off of LSM for AATD liver disease. In a study including 94 Pi*ZZ patients with paired LSM and liver biopsies, Clark et al. [26] observed that cut-offs of 5.54 and 8.45 kPa had the highest accuracy for detecting significant fibrosis (≥F2) and advanced fibrosis (≥F3), respectively. However, these cut-offs had a low specificity and a low positive predictive value. Hamesch et al. [17] increased the cut-off for significant fibrosis to >7.1 kPa in order to increase the positive predictive value, confirming the presence of ≥F2 in 22 out of 23 patients with liver biopsies [27], while Guillaud et al. [16] suggested an LSM > 7.2 kPa for significant fibrosis and LSM > 14 kPa for cirrhosis. In another study in 75 patients with AATD, the investigators offered a liver biopsy to all individuals with a LSM > 6 or altered liver enzymes in combination with an abnormal ultrasound. Among the 11 biopsies analyzed, they found that the LSM scores in patients with moderate or severe fibrosis were >8 kPa [18]. According to these results and the cut-offs previously established in other etiologies, we chose an arbitrary cut-off of LSM > 7.5 kPa as suggestive of significant fibrosis, and LSM ≥ 10 kPa as advanced fibrosis/cirrhosis. In our sample, there were two Pi*SZ patients with LSM = 7.3 kPa, one of whom was overweight and had diabetes mellitus and increased GGT values, and the other was a Pi*MZ patient with LSM = 7.5 kPa without other identified risk factors of liver disease. Since the etiology of liver disease has an impact on LSM and the data on AATD induced liver disease are limited [28], further studies are needed to validate the best LSM cut-off for screening of liver disease in AATD.

Ten percent of Pi*ZZ patients in our cohort had LSM > 7.5 kPa, similar to the prevalence of liver fibrosis reported in initial studies in AATD patients, which varied from 10–15% in clinical studies [29,30] to 37% in autopsy studies [31]. More recently, with the development of transient elastography, there has been growing interest in the early detection of liver disease in AATD. The study by Guillaud et al. [16] described 5 patients (18%) with LSM suggestive of significant fibrosis and 2 patients (7%) with LSM suggestive of advanced liver fibrosis/cirrhosis. Other studies have reported a higher prevalence; Hamesch et al. [17] described a prevalence of liver fibrosis of 23.6% among 403 Pi*ZZ individuals and observed that liver disease was 9 to 20 times more frequent in this population compared to non-AAT-deficient individuals. In a cohort of COPD Pi*ZZ patients referred for lung transplantation, Morer et al. [32] found that 13% of patients had significant fibrosis (F2) and 8% advanced fibrosis (≥F3). Similar to these numbers, 8 (14%) of our COPD Pi*ZZ patients had LSM > 7.5 kPa, while in 3 (5.7%) LSM was higher than 10 kPa, suggesting the presence of advanced fibrosis.

In our cohort, Pi*MZ individuals had lower values of LSM compared to Pi*ZZ individuals. The mean LSM was 4.7 kPa for the 34 Pi*MZ patients included. None of these patients had values above 7.5, and only one had LSM = 7.5 kPa. In this patient, other co-factors for liver disease such as obesity, alcohol consumption, or metabolic syndrome were not found. The incidence of liver disease could be higher in heterozygous Z than in the general population, although some authors have hypothesized that while the Pi*MZ genotype acts as a disease modifier, it is not sufficient per se to trigger clinically relevant liver impairment [33]. In a study that analyzed 1184 individuals with non-alcoholic fatty liver disease (NAFLD) and 2462 with chronic alcohol misuse, the Z variant increased the risk of patients with NAFLD to develop cirrhosis and was more frequently present in alcohol misusers with cirrhosis compared to those without significant liver injury [34]. In contrast, a recent analysis of data from the European alpha-1 liver cohort showed that 10% out 419 Pi*MZ had LSM values ≥ 7.1 kPa compared with 4% of non-Z carriers. After adjusting for potential confounders, Pi*MZ individuals still had significantly higher odds for LSM ≥ 7.1 kPa [12]. There is agreement that, in coexistence with other risk factors, and especially in the context of alcohol misuse or NAFLD, Z carriage is a strong risk factor for the development of cirrhosis [17,18] and may also lead to faster hepatic decompensations [35]. In our cohort, 60% of Pi*ZZ patients with LSM > 7.5 kPa had some alcohol consumption and had a higher BMI than those with LSM ≤ 7.5 kPa, and, therefore, these factors could have contributed to the progression of liver disease.

Liver enzymes have often been used to screen liver disease in AATD in clinical practice [36]. In our cohort, elevated liver enzymes and FIB-4 were more frequently observed in patients with LSM > 7.5 kPa, but normal levels were also frequently present in patients with high LSM. In fact, liver enzyme alterations ranged from only 25% of cases for AST and ALT to 37.5% for GGT in Pi*ZZ patients with LSM > 7.5 kPa. Patients with fibrosis or even cirrhosis may present normal serum liver enzymes [11], and this has also been observed in Pi*ZZ individuals [13,17]. On the other hand, up to 10% of AATD patients with normal liver function tests and ultrasound may have increased LSM values [16]. Furthermore, an increase in ALT has a low sensitivity for identifying liver disease in AATD individuals [13,15]. In the European alpha-1 liver cohort, heterozygous Pi*MZ carriers also had higher serum transaminases compared to non-carriers, although this percentage varied from 5.4% to 28.6% and was higher in individuals older than 50 years [12].

The relationship between lung and liver disease in individuals with AATD is controversial. The first series of patients with the deficiency suggested that lung and liver disease rarely coexisted in AATD, and liver disease was more frequently reported in AATD never smokers compared to smokers [37,38]. However, more recent studies using new diagnostic techniques have reported more frequent coexistence of the alterations in both organs [39]. In this line, all of our patients with elevated LSM also had COPD, although the correlation between lung function and LSM was not significant. Moreover, recruiting patients from respiratory departments may have influenced the high prevalence of COPD among patients with elevated LSM; although they were also older, with higher BMI and with a higher frequency of alcohol misuse compared with patients with normal LSM. Therefore, a clear relationship between elevated LSM and lung disease cannot be established from our results.

Our study had some limitations. First, the identification of liver fibrosis was only made by transient elastography as we did not perform liver biopsies. However, as there are no specific treatments for AATD liver disease to date, the performance of an invasive diagnostic technique in otherwise asymptomatic patients may not be justified. Second, this was a cross-sectional study, and data on the evolution of LSM over time were not available. Third, the design of our study did not allow us to investigate a causal relationship between AATD and liver alterations. Our sample size was not big enough for a multivariate analysis adjusted for known confounders of increased liver fibrosis. However, the study had some strengths: We recruited individuals from three reference centers, and, considering that AATD is a rare disease, we reported information from a large series of patients with homozygous and heterozygous AATD.

In conclusion, the results of this study support the assessment of liver disease in all AATD Pi*ZZ individuals and heterozygous Pi*Z individuals with additional liver risk factors. Transient elastography has been shown to be a valuable tool to screen for AATD liver disease, and collaboration between hepatologists and pneumologists is crucial for providing the best care to AATD patients. Due to the poor correlation between liver enzymes and other serum biomarkers and the underlying liver disease, all Z-allele carriers, even those with normal serum biomarker values, should be screened with transient elastography. Since AATD is a rare disease, international collaboration in large registries is needed to investigate the best screening strategy for lung and liver disease [12,17,40].

## Figures and Tables

**Table 1:** Baseline characteristics of the patients included by AAT genotype.

	ZZ (*n* = 81)	Heterozygous Z (*n* = 67)	*p*-Value
Age	57.0 (14.4)	52.5 (14.5)	0.051 ^1^
Sex, men	41 (50.6%)	34 (50.7%)	0.985 ^2^
BMI	25.1 (3.9)	24.0 (7.0)	0.398 ^1^
Smoking exposure:			0.010 ^2^
Active	43 (53.1%)	22 (32.8%)	
Former smoker	7 (8.6%)	16 (23.9%)	
Never smoker	31 (38.3%)	29 (43.3%)	
Alcohol consumption	19 (23.5%)	19 (28.4%)	0.991 ^2^
Diabetes mellitus	0 (0%)	2 (3.0%)	0.203 ^2^
Hypertension	14 (17.5%)	16 (23.9%)	0.453 ^2^
AAT levels, mg/dL	33.3 (61.9)	71.9 (20.8)	<0.001 ^1^
Reason for diagnosis:			0.002 ^2^
Liver disease	9 (11.1%)	13 (19.4%)	
Lung disease	52 (64.2%)	23 (34.3%)	
Family study	17 (21.0%)	28 (41.8%)	
Other	3 (3.7%)	3 (4.5%)	
COPD	57 (70.4%)	15 (22.7%)	<0.001 ^2^
Asthma	5 (7.8%)	14 (21.2%)	0.056 ^2^
Neonatal jaundice	6 (7.4%)	3 (4.5%)	0.513 ^2^
FVC, L	3.6 (1.5)	3.9 (1.1)	0.197 ^1^
FVC, %	90.0 (28.5)	99.8 (19.8)	0.033 ^1^
FEV1, L	2.1 (1.2)	3.0 (1.2)	<0.001 ^1^
FEV1, %	69.0 (30.5)	92.9 (27.6)	<0.001 ^1^
FEV1/FVC	0.6 (0.2)	0.7 (0.2)	0.001 ^1^
KCO, %	51.0 (32.5)	58.9 (36.7)	0.231 ^1^

Footnote: BMI: Body mass index; COPD: Chronic obstructive pulmonary disease; FVC: Forced ventilatory capacity; FEV1: Forced expiratory volume in 1 s; KCO: Transfer coefficient of the lung for carbon monoxide; AAT: Alpha-1 antitrypsin. ^1^ Mann–Whitney U-test *p*-value, ^2^ Chi-squared *p*-value.

**Table 2:** Results of blood analysis and transient elastography in patients with different AAT genotypes.

	ZZ (*n* = 81)	Heterozygous Z (*n* = 67)	*p*-Value
Laboratory findings			
Platelet count, ×10^9^/L	222 (59)	239 (61)	0.074 ^1^
INR	1.0 (0.2)	1.0 (0.1)	0.067 ^1^
Bilirubin, mg/dL	0.8 (0.5)	0.7 (0.3)	0.158
AST, IU/L	29.2 (15.4)	25.0 (8.0)	0.029 ^1^
AST > ULN	4 (4.9%)	4 (6%)	0.869 ^2^
ALT, IU/L	26.6 (22.6)	26.1 (13.4)	0.967 ^1^
ALT > ULN	6 (7.4%)	5 (7.5%)	0.952 ^2^
ALP, IU/L	78.2 (29.6)	81.8 (21)	0.412 ^1^
ALP > ULN	6 (7.4%)	2 (3%)	0.294 ^2^
GGT, IU/L	36.2 (33.9)	31.1 (29.4)	0.336 ^1^
GGT > ULN	13 (16.5%)	9 (13.6%)	0.637 ^2^
Albumin, g/dL	4.3 (0.6)	4.4 (0.3)	0.044 ^1^
Cholesterol, mg/dL	207 (35)	198 (36)	0.161
FIB-4	1.6 (0.8)	1.2 (0.5)	<0.001
FIB-4 < 1.45	38 (47.5%)	51 (78.5%)	<0.001 ^2^
FIB-4 > 3.25	5 (6.2%)	0	0.065 ^2^
APRI	0.35 (0.18)	0.27 (0.09)	<0.001 ^1^
APRI < 0.5	67 (83)	64 (91)	0.023 ^2^
APRI > 1.0	1 (1.2)	0	0.956
ELF, n = 60	8.6 (0.8)	8 (0.6)	0.007 ^1^
Transient elastography			
LSM	5.6 (2.4)	4.6 (1.2)	0.001 ^1^
LSM > 7.5 kPa	8 (9.9%)	0	0.040 ^2^
LSM ≥ 10 kPa	3 (3.7%)	0	
CAP	256 (59)	253 (50)	0.252 ^1^
CAP 268–280 dB/m	7 (8.6%)	4 (6%)	0.807 ^2^
CAP > 280 dB/m	26 (32.1%)	21 (31.3%)	

Footnote: INR: International normalized ratio; ULN: Upper limit of normal; AST: Aspartate aminotransferase; ALT: Alanine aminotransferase; ALP: Alkaline phosphatase; GGT: Gamma-glutamyl transferase; FIB-4: Fibrosis 4; APRI: AST to platelet ratio index; ELF: Enhanced liver fibrosis; LSM: Liver stiffness measurement; CAP: Controlled attenuation parameter. ^1^ Mann–Whitney U-test *p*-value, ^2^ Chi-squared *p*-value.

**Table 3:** Comparison between Pi*ZZ individuals based on liver stiffness (LSM) and diagnosis of chronic obstructive pulmonary disease (COPD).

	LSM ≤ 7.5 (*n* = 73)	LSM > 7.5 (*n* = 8)	*p*-Value	No COPD (*n* = 24)	COPD (*n* = 57)	*p*-Value
Age	56.2 (14.5)	64.9 (11.4)	0.076	46.2 (14.5)	61.6 (11.7)	<0.001 ^1^
Sex, men	37 (50.7%)	4 (50%)	1.00	11 (45.8%)	30 (52.6%)	0.752 ^2^
BMI	24.6 (3.4)	29.0 (5.3)	0.056	24.2 (3.7)	25.4 (3.9)	0.186 ^1^
Smoking exposure:			0.527			<0.001 ^2^
Active	37 (50.7%)	6 (75%)		7 (29.2%)	36 (63.2%)	
Former smoker	7 (9.6%)	0 (0%)		0 (0%)	7 (12.3%)	
Never smoker	29 (39.7%)	2 (25%)		17 (70.8%)	14 (24.6%)	
Alcohol consumption	16 (24.2%)	3 (60%)	0.115	5 (25%)	14 (27.2)	1.000 ^2^
Hypertension	10 (13.9%)	4 (50%)	0.028	1 (4.2%)	13 (23.2%)	0.054 ^2^
COPD	49 (67.1%)	8 (100%)	0.097	0	57 (100%)	0.001 ^2^
Neonatal jaundice	6 (8.2%)	0 (0%)	1.000	4 (16.7%)	2 (3.5%)	0.060 ^2^
FEV1, %	70.4 (30.7)	56.4 (27.3)	0.205	99.6 (13.1)	56.2 (26.3)	<0.001 ^1^
Laboratory findings:						
Platelet count, ×10^9^/L	224 (60)	202 (49)	0.267	210 (48)	226 (62)	0.214 ^1^
INR	1.0 (0.2)	1.1 (0.1)	0.378	1.0 (0.1)	1.1 (0.2)	0.040 ^1^
Bilirubin, mg/dL	0.8 (0.5)	0.6 (0.2)	0.262	1.0 (0.9)	0.7 (0.2)	0.143 ^1^
AST, UI/L	27.2 (10.1)	47.6 (34.7)	0.141	27.2 (10.6)	30.0 (16.9)	0.375 ^1^
AST > ULN *	2 (2.7%)	2 (25%)	0.048	1 (4.2%)	3 (5.3%)	0.675 ^2^
ALT, UI/L	24.2 (14.2)	48.8 (55.8)	0.254	25.5 (14.4)	27.1 (25.3)	0.719 ^1^
ALT > ULN *	4 (5.5%)	2 (25.0%)	0.108	3 (12.5%)	3 (5.3%)	0.226 ^2^
ALP, UI/L	78.5 (30.9)	75.9 (14.8)	0.816	70.3 (31)	81.4 (28.7)	0.130 ^1^
ALP > ULN *	6 (8.5%)	0 (0%)	1.000	2 (8.3%)	4 (7.0%)	1.000 ^2^
GGT, UI/L	31.8 (19.3)	75.6 (84.1)	<0.001	33.2 (22.2)	37.2 (37.7)	0.685 ^1^
GGT > ULN *	10 (14.1%)	3 (37.5%)	0.120	5 (20.8%)	8 (14%)	0.589 ^2^
Albumin, g/dL	4.3 (0.6)	4.4 (0.3)	0.615	4.5 (0.3)	4.2 (0.6)	0.004 ^1^
Cholesterol, mg/dL	206 (35)	208 (39)	0.901	205 (39)	207 (34)	0.824 ^1^
FIB-4	1.5 (0.8)	2.2 (0.7)	0.032	1.3 (0.8)	1.7 (0.8)	0.046 ^1^
FIB-4 < 1.45:	37 (50.7%)	1 (12.5%)	0.059	15 (62.5%)	23 (40.4%)	0.077 ^2^
FIB-4 > 3.25:	4 (5.5%)	1 (12.5%)	0.418	1 (4.2%)	4 (7%)	1.000 ^2^
APRI	0.33 (0.1)	0.56 (0.3)	<0.001	0.35 (0.17)	0.35 (0.19)	0.992 ^1^
Transient elastography						
LSM	5.0 (1.1)	10.8 (4.6)	0.009	5.3 (1.1)	5.7 (2.8)	0.361 ^1^
CAP	249 (56)	318 (48)	0.004	233 (56)	266 (58)	0.023 ^1^
LSM > 7.5 kPa:	0	8 (100%)	NA	0	8 (14.0%)	0.097 ^2^

Footnote: BMI: Body mass index; COPD: Chronic obstructive pulmonary disease; FEV1: Forced expiratory volume in 1 s; AAT: Alpha-1 antitrypsin; INR: International normalized ratio; AST: Aspartate aminotransferase; ALT: Alanine aminotransferase; ALP: Alkaline phosphatase; GGT: Gamma-glutamyl transferase; ULN: Upper limit of normal; FIB-4: Fibrosis 4; APRI: AST to platelet ratio index; ELF: Enhanced liver fibrosis; LSM: Liver stiffness measurement; CAP: Controlled attenuation parameter. *: Upper limit of normal according to sex-specific cut-offs: For AST and ALT: >35 IU/L in female, >50 IU/L in male; for ALP: >120 IU/L for both genders; for GGT: >38 IU/L in females and >55 IU/L in males. ^1^ Mann–Whitney U-test *p*-value, ^2^ Chi-squared *p*-value.

## Data Availability

Data are available from the authors upon request.
